# Comparison of the effects of fentanyls and other *μ* opioid receptor agonists on the electrical activity of respiratory muscles in the rat

**DOI:** 10.3389/fphar.2023.1277248

**Published:** 2023-11-23

**Authors:** Damiana Cavallo, Eamonn Kelly, Graeme Henderson, Ana Paula Abdala Sheikh

**Affiliations:** School of Physiology, Pharmacology and Neuroscience, University of Bristol, Bristol, United Kingdom

**Keywords:** wooden chest syndrome (WCS), fentanyl, opioids, respiratory depression, muscle rigidity

## Abstract

**Introduction:** Deaths due to overdose of fentanyls result primarily from depression of respiration. These potent opioids can also produce muscle rigidity in the diaphragm and the chest muscles, a phenomenon known as Wooden Chest Syndrome, which further limits ventilation.

**Methods:** We have compared the depression of ventilation by fentanyl and morphine by directly measuring their ability to induce muscle rigidity using EMG recording from diaphragm and external and internal intercostal muscles, in the rat working heart-brainstem preparation.

**Results:** At equipotent bradypnea-inducing concentrations fentanyl produced a greater increase in expiratory EMG amplitude than morphine in all three muscles examined. In order to understand whether this effect of fentanyl was a unique property of the phenylpiperidine chemical structure, or due to fentanyl’s high agonist intrinsic efficacy or its lipophilicity, we compared a variety of agonists with different properties at concentrations that were equipotent at producing bradypnea. We compared carfentanil and alfentanil (phenylpiperidines with relatively high efficacy and high to medium lipophilicity, respectively), norbuprenorphine (orvinolmorphinan with high efficacy and lipophilicity) and levorphanol (morphinan with relatively low efficacy and high lipophilicity).

**Discussion:** We observed that, agonists with higher intrinsic efficacy were more likely to increase expiratory EMG amplitude (i.e., produce chest rigidity) than agonists with lower efficacy. Whereas lipophilicity and chemical structure did not appear to correlate with the ability to induce chest rigidity.

## 1 Introduction

Fentanyl and its structural analogues (commonly referred to as “fentanyls”) are currently responsible for most illicit opioid overdose deaths in North America and Canada, (British Columbia Coroners Services, 2022; Centers for Disease Control and Prevention, 2022). In the United States they have been reported to be involved in more than 66% of drug deaths in 2021, compared to only 18% in 2015. Fentanyl is a synthetic *μ* opioid receptor agonist that has 50 to 100 times higher potency than morphine to depress respiration (Harper et al., 1976). The primary cause of death from opioid overdose is hypoxia (Solis et al., 2017; Curay et al., 2023) due to respiratory depression, characterized by bradypnea and a decrease in chemosensory drive (White and Irvine, 1999; Pattinson, 2008). Opioid-induced respiratory depression results from activation of *μ* opioid receptors in the brainstem respiratory centres (Dahan et al., 2001; Pattinson, 2008). Following i. v. administration, fentanyls rapidly depress respiration (Stoeckel et al., 1982; Varshneya et al., 2022). Their high lipid solubility enables them to rapidly penetrate the brain and thus reach overdose levels more quickly than heroin (Darke and Duflou, 2016), reducing the opportunity for intervention (Skolnick, 2018).

Fentanyls induce skeletal muscle rigidity (stiffness) by increasing motor neuron output from the central nervous system (Willette et al., 1982; Lui et al., 1989; Campbell et al., 1995). This results from *μ* opioid receptor activation at various sites in the brain (Weinger et al., 1991; Vankova et al., 1996). When such rigidity is observed in the respiratory muscles of the chest wall in humans - diaphragm and intercostals - it produces a phenomenon referred to as Wooden Chest Syndrome (WCS) (Scamman, 1983; Campbell et al., 1995; Fahnenstich et al., 2000). Fentanyls also induce contraction of upper airway muscles causing laryngospasm in rats (Willette et al., 1982; Willette et al., 1987; Saunders et al., 2022). These effects on upper airway and chest wall respiratory muscles further reduce ventilation (Çoruh et al., 2013; Burns et al., 2016). In animal models, non-fentanyl opioid agonists, such as morphine and etonitazine, have also been observed to induce skeletal muscle rigidity, although their specific effects on respiratory muscles were not reported (Rackham, 1980; Slater and Starkie, 1987). Similarly, the selective *μ* opioid peptide agonist [D-Ala^2^, N-MePhe^4^, Gly-ol]-enkephalin (DAMGO) can induce skeletal muscle rigidity but only when microinjected directly into the brain, presumably because its low lipid solubility does not allow it to penetrate the blood-brain barrier (Widdowson et al., 1986; Slater and Starkie, 1987; Vankova et al., 1996).

Naloxone is currently the main pharmacological agent used to reverse opioid overdose. However, it has some limitations: for example, respiratory depression induced by high potency agonists has been reported to show reduced sensitivity to reversal by naloxone (Schumann et al., 2008; Peterson et al., 2016; Fairbairn et al., 2017; Lynn and Galinkin, 2018; Gill et al., 2019; Hill et al., 2019). Higher doses of naloxone can reverse fentanyl-induced respiratory depression, but with some disadvantages, e.g., it can precipitate opioid withdrawal and its short duration of action means that re-overdosing may occur if the respiratory depressant effect of the opioid agonist outlasts the naloxone antagonism (Levine et al., 2020; van der Schrier et al., 2022).

In the current study, our main objective was to investigate the impact of various opioid agonists on the electrical activity of respiratory muscles, specifically focusing on muscle rigidity. We specifically explored the concentrations of these agonists that resulted in a comparable decrease in respiratory rate of approximately 40%, by studying a range of opioids that differ in chemical structure, lipid solubility and agonist intrinsic efficacy. To compare their ability to induce rigidity in different respiratory muscles, we used the *in situ* decerebrated and arterially perfused rat preparation, referred to as the working heart-brainstem preparation (WHBP) (Paton, 1996; Levitt et al., 2015). This preparation allows the simultaneous measurement of electromyographic (EMG) activity in the diaphragm and external and internal intercostal muscles as an indicator of muscle rigidity. During the initial phase of our research, we conducted preliminary experiments to ascertain the concentration of each drug that induced approximately 40% depression of respiration. We based this target on the same level of respiratory depression that we had previously studied in awake, freely moving mice in plethysmography experiments (Hill et al., 2019). The respiratory rate was measured from the phasic pattern of diaphragm muscle activity. This preparation displays a fictive eupnoeic (i.e., normal) respiratory pattern generated by an intact brainstem respiratory circuitry with preserved outputs to motor nerves. Furthermore, anaesthesia is absent and respiratory gases (O_2_ and CO_2_) are clamped and independent of changes in ventilation. This eliminates any confounding effects from anaesthetic-induced respiratory depression and reflex respiratory responses to shifts in blood gases (Paton, 1996). We also examined the ability of fentanyl to induce changes in upper airway resistance by measuring changes in subglottal pressure under a constant airflow (Dutschmann and Herbert, 2006).

## 2 Materials and methods

### 2.1 Animals

Male rats (Wistar; Charles River or Envigo) aged between P21 and P28 (75–100 g) (Paton et al., 2022), were used for all experiments. The choice to use pre-pubescent males in our study was primarily driven by a demographic consideration. In Northern American approximately 70% of individuals who succumbed to opiate toxicity were male. Therefore, our decision to include males aligns with the real-world distribution of overdose cases in these regions, allowing us to better represent the population affected by this issue. We acknowledge that our choice of pre-pubescent males may not directly correlate with the age group mentioned drug overdose statistics in the introduction, which are largely related to young adults. However, the age group selected for our study was determined based on practical and experimental limitations related to model (for a comprehensive review see Paton et al. (2022). Rats were group housed on a 12 h light-dark cycle at 22°C with *ad libitum* access to food and water. All procedures were performed in accordance with the United Kingdom Animals (Scientific Procedures) Act 1986, the European Communities Council Directive (2010/63/EU) and the University of Bristol ethical review document.

### 2.2 Drugs

Alfentanil hydrochloride (LGC Standards, London, United Kingdom), carfentanil (Cayman Chemical Company, MI, United States), fentanyl citrate, levorphanol tartrate, naloxone hydrochloride (all from Sigma Aldrich, Dorset, United Kingdom), morphine hydrochloride (Macfarlan Smith, Edinburgh, United Kingdom), norbuprenorphine (National Institute on Drug Abuse, MD, United States), vasopressin (Tocris, United Kingdom), heparin (5000 I.U./ml, Wockhardt, United Kingdom) and vecuronium bromide (Sigma Aldrich, United States) were each dissolved in saline.

### 2.3 *In situ* experiments

Electromyographic (EMG) recordings of muscle activity from diaphragm, external and internal intercostal muscles were performed using the rat *in situ* arterially perfused working heart-brainstem preparation (Paton, 1996; Levitt et al., 2015). Rats were heparinized (1000 U, I.P.) and 20 min later were deeply anaesthetized with isoflurane 5% in 100% O_2_ until the respiration was severely depressed and the animal failed to respond to noxious pinch of tail or toe. Rats were bisected below the diaphragm and decerebrated at the precollicular level in ice-cold modified Ringer solution containing (in mM): 125 NaCl, 3.95 KCl, 2.5 CaCl_2_, 1.25 MgCl_2_, 1.25 KH_2_PO_4_, 10 D-glucose and 25 NaHCO_3_. The cerebellum was left intact. The lungs were removed by opening the right half of the diaphragm along its rib insertion. Bipolar EMG electrodes were placed in the intercostal and diaphragm muscles as described below.

The preparation was then transferred to a recording chamber in the supine position, a double lumen catheter was placed in the descending aorta and perfused, using a peristaltic pump (Watson-Marlow 505S; flow rate 21–24 mL min^−1^), with warmed (30°C–32°C) modified Ringer solution containing polyethylene glycol MW 20,000 (1.25%) to maintain oncotic pressure and bubbled with 95% O_2_/5% CO_2_. The perfusate was filtered and passed through bubble traps to remove gas bubbles. The second lumen of the catheter output was connected to a pressure transducer (BPM-832 Pressure Monitor, CWE, Inc., Ardmore, PA 19003, United States) that allowed for monitoring and maintenance of perfusion pressure (50–70 mmHg). Vasopressin (200–400 pM) was added to the perfusate to induce peripheral vasoconstriction, increase brain perfusion and stabilize perfusion pressure.

Once respiratory output as observed in the diaphragm EMG was robust and stable, 10 min of baseline activity was recorded. Then, a single concentration of opioid agonist was applied in the perfusion solution until the bradypnea reached a steady state (usually between 2.5 and 3 min). Subsequently, naloxone (1 µM) was applied in the perfusion solution to reverse the effects of the opioid agonists. We have chosen to use this high concentration as fentanyls have been reported to be less susceptible to naloxone reversal than other opioids such as morphine (see Introduction), but it is unlikely that non-opioid effects of naloxone would be seen at this concentration (Dingledine et al., 1978). At the end of the experiment, vecuronium bromide (4 μg/mL) was added to the perfusate to block neuromuscular transmission. This addition served three primary purposes. Firstly, it allowed us to establish the baseline noise level in each electrode. Secondly, it confirmed if the observed agonist-mediated muscle effects remained unaffected by factors downstream of the neuromuscular junction. Thirdly, in the case of subglottal pressure experiments, vecuronium bromide was employed to ascertain the residual level of subglottal pressure in instances where laryngeal muscle activity was fully relaxed, thereby enabling a precise assessment of glottal function in the presence of *μ* opioid receptor agonists compared to its resting state. The animals were randomised in a block design where each animal in a block of viable preparations was randomised to a single concentration of an opioid or vehicle.

### 2.4 EMG recording

Bipolar EMG electrodes, were constructed using a previously described method (Pearson et al., 2005), that we customized for our needs. They were made from Teflon coated annealed stainless-steel wire (Silver wire, temper annealed, quadruple PTFE insulated, ∅ 0.02 mm, coating thickness 0.035 mm, Advent; Silver insulated wire, ∅ 0.125 mm, insulation thickness 0.0125, Goodfellow, United Kingdom). Three pairs of electrodes were prepared as follows. For each electrode pair, two pieces of wire were tightly twisted together, and a suture knot was placed and fixed with epoxy, about 4–6 cm from one end of the pair. A few millimetres from the knot, approximately 1 mm of the Teflon insulation was removed from each wire so that the two bared regions were separated by about 2 mm. The ends of the two twisted wires were crimpled into the shaft of a needle. The opposite ends of the wires were cut about 3–4 cm from the knot and bared. The needle attached to the end of each pair of electrodes was used to draw the twisted pair of wires through the muscle until the knot proximal to the bared regions was firmly against the surface of the muscle. The two distal ends of the electrode pair exiting the muscle were connected to the amplifier through bipolar cables. Electrodes were placed on the external left-hand side of the rib cage for the external intercostals, on the internal left-hand side of the rib cage for the internal intercostals, usually keeping the same position between the ribs, and on the right-hand side of the diaphragm close to the rib cage for diaphragm. EMG recordings were AC amplified (x 1 k for diaphragm and x 10 k for intercostal muscles, Amplifier 1700, A–M Systems, Carlsborg, WA, United States), band pass filtered (300 Hz–5 kHz) and digitized at 5 kHz (AD converter and Spike2 software, Cambridge Electronic Design, Cambridge, United Kingdom). Electromyograms were positively rectified and integrated (time constant = 50 ms).

### 2.5 Subglottal pressure measurements

In a separate series of experiments, changes in airflow resistance through the larynx were inferred by measuring subglottal pressure (SGP), under a constant airflow, in the *in situ* arterially perfused working heart–brainstem preparation. The trachea below the larynx was cannulated in the direction of the pharynx with a T-shaped catheter. Through one arm of this cannula a constant flow of warmed (31°C) and humidified carbogen gas was passed in the expiratory direction (Paton et al., 1999). SGP was recorded from the other arm of the T-shaped cannula using a custom-built pressure transducer**.** The ventral pharyngeal wall just above the larynx was opened to allow the gas to escape without added resistance from the oropharynx. Increases and decreases in SGP were indicative of constriction (adduction) and dilation (abduction), respectively, thereby giving an index of the dynamic changes in upper airway resistance during the respiratory cycle. The rate of airflow through the larynx was set between 50 and 60 mL/min (a modification of Dutschmann and Paton, 2005). Respiratory motor output was recorded from the diaphragm through a bipolar EMG electrode as described above.

### 2.6 *In vitro* G protein activation assay

HEK 293 cells were cultured at 37°C in DMEM supplemented with 10% fetal bovine serum and penicillin/streptomycin. Cells were seeded onto 10 cm dishes and grown to 80% confluency before transfection. To determine the relative ability of the opioid agonists to activate Gi proteins, a bioluminescence resonance energy transfer (BRET^2^) based assay that monitors the separation of Gαi1 and Gγ2 was used (a modification of Hill et al., 2018). HEK 293 cells were transiently transfected with rat HA-tagged *μ* receptors, Gαi1-Renilla luciferase II (RlucII) and GFP10-Gγ2. Immediately prior to each assay, cells were resuspended in clear DMEM and then transferred to a 96 well plate at 90 μL per well. Measurements of BRET were made at 37°C. Coelenterazine 400a, at a final concentration of 5 μM, was injected 5 s prior to reading the cell plate. BRET measurements were made on a FLUOstar Omega plate reader (BMG LABTECH, Ortenberg, Germany) using the following filter set: acceptor, 515 ± 30 nm; and donor, 410 ± 80 nm. BRET signals were determined as the ratio of the light emitted by acceptor (GFP10) over donor (RlucII). For Gi activation, BRET measurements were taken 2 min after agonist application. Agonist stimulation resulted in a rapid decrease in the BRET signal between Gαi1-RlucII and GFP10-Gγ2 in cells co-expressing *μ* receptors.

### 2.7 Data analysis

**FIGURE 1:**
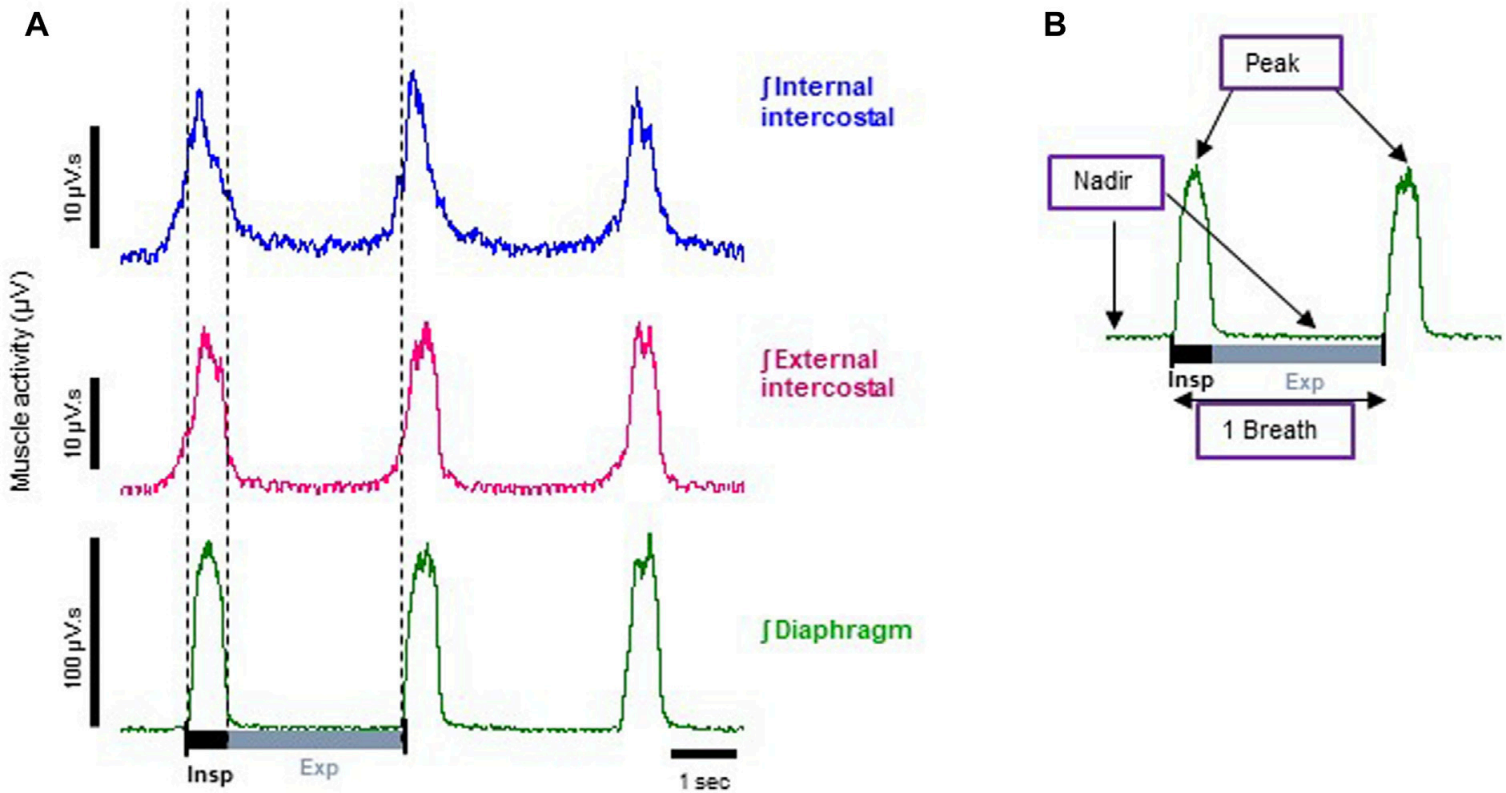
Representative electromyographic (EMG) traces recorded from the arterially perfused working heart-brainstem preparation of the rat. **(A)** Typical integrated (ʃ) EMG recordings from diaphragm (green trace), external (magenta trace) and internal (blue trace) intercostal muscles. Each trace shows phasic respiratory activity. Note: the bursts that appear in the blue trace during inspiration are the result of external intercostal muscle activity, as the signal bleeds through due to these muscles being very thin and in contact to each other. For the same reason, the small increase in electrical activity before the inspiration burst shown in the external intercostal signal (magenta trace) is bleed through from the internal intercostal muscle which is active in the late expiratory phase (known as pre-inspiration). **(B)** A typical respiratory cycle (inspiration + expiration = 1 breath) from the diaphragm EMG trace shown in **A**, whose phasic frequency has been taken as the measure of respiratory rate (breaths per minute). Indicated on the trace are the parameters we measured: nadir, which is the minimum inter-burst interval, and peak, which is the peak burst amplitude of the EMG signal.

*EMG recording experiments.* Respiratory parameters were determined from integrated diaphragm, external and internal intercostal muscle electrical activity signals (see Figure 1) using a custom written algorithm (Abdala Sheikh, 2021). Respiratory rate was determined as number of bursts per minute, from the diaphragm bursts. Minimum EMG amplitude during expiration (Nadir) and maximum EMG amplitude during inspiration (Peak) were calculated for each burst, for each muscle, and measured as μV. The ratio of nadir to peak (Nadir/Peak [*100]) was calculated. For each experiment, parameters were averaged in 30 s bins, and the subsequent change in each parameter following drug administration calculated as a percentage of the pre-drug baseline (set to 100); for the ratio of the nadir to peak, the mean baseline was set to 1. This value represents the ratio between NADIR and PEAK, quantifying the magnitude of the nadir relative to the peak. This ratio provides insight into the extent of tidal volume reduction that would occur in an intact animal. In this context, “Nadir”, expressed in μV, denotes the lowest breath-by-breath biopotential recorded during the diaphragm’s relaxation phase (expiration). Conversely, “Peak” refers to the highest breath-by-breath biopotential measured during the diaphragm’s contraction phase (inspiration) in relation to an average baseline noise level established after vecuronium bromide administration at the conclusion of the experiment. Therefore, they are indeed different from a conventional breath by breath hight. For each experimental group (*n* = 5 for each opioid agonist), the overall change of each parameter was calculated from the average of data points at steady state. In carfentanil and norbuprenorphine treated mice, we observed that the maximum respiratory depression occurred at later time points during agonist exposure than with other agonists. To determine the overall change in measured outputs, we calculated the average of data points that reached the lowest respiratory rate for each drug. In the cases where the presence of opioid agonist caused a switch from the eupnoea (3-phase respiratory cycle) to apneusis (2-phase respiratory cycle) (Smith et al., 2007), resulting in an increase in respiratory rate, we excluded those specific data points from the averages. Additionally, the time profile of each opioid agonist’s response varied slightly across all the experiments, and thus, the addition of naloxone (an opioid receptor antagonist) did not occur at the same time in each experiment. To address these variations, we chose to interrupt the *X*-axis to align the interventions for each experiment, making the comparison more consistent and accurate. Outliers were identified by using the Rout method of GraphPad Prism (version 8, GraphPad Software Inc., San Diego, CA). Statistical comparisons of grouped data were made using one-way ANOVA with Dunnet’s multiple comparison test using GraphPad Prism (version 8). Data in time course graphs are expressed as mean ± SEM. Data in the bar graphs are expressed as mean ± SD.

*Subglottal measurements.* In a separate series of experiments (*n* = 6), changes in airflow resistance were measured from recordings of SGP, using a custom written algorithm (Abdala Sheikh, 2021). For each respiratory cycle, marked by the diaphragm EMG activity, we measured the minimum SGP value (Trough), corresponding to the inspiratory decrease in glottal resistance, and the maximum SGP value (Peak), corresponding to the post-inspiratory increase in glottal resistance. In the presence of fentanyl or vecuronium bromide, when the changes in both peaks and troughs reached a steady state converging to an SGP level halfway between the two, we measured the SGP values from a sample of trace at steady state and averaged them. Statistical comparisons of grouped data were made using one-way ANOVA with Šídák’s multiple comparisons test using GraphPad Prism (version 8). Data in the bar graphs are presented as mean ± SD.

We also performed a qualitative analysis of changes in SGP by visual inspection of the SPG trace (see example in Figure 9D) to highlight increases in SGP during inspiration as our quantitative analysis would not have taken these into account.

*G protein activation assay*. Agonist concentration-response data were fitted to sigmoidal curves with variable slopes, with the bottom of the curve in each case being constrained to 0 using GraphPad Prism (version 8). Because the agonists each have different maximum responses, to compare their potencies we measured relative potency at a single BRET ratio level (0.075) that is submaximal for all agonists (see Figure 6).

## 3 Results

### 3.1 EMG recording from respiratory muscles in the arterially perfused working heart–brainstem preparation

Typical control EMG recordings from diaphragm, external and internal intercostal muscles obtained from the arterially perfused working heart-brainstem preparation of the rat are shown in Figure 1. On the left are representative traces of respiratory phasic EMG activity from each of the three muscles (raw experimental traces are shown in Sup. Figure 1). The diaphragm functions as the inspiratory pump and is active mainly during inspiration (burst of muscle activity in the trace) (Dutschmann and Dick, 2012), during which the muscle contracts and lowers, thus increasing the volume of the rib cage. During expiration, instead, it passively relaxes (inter-burst interval), completing the respiratory cycle (one breath). Its phasic frequency has been taken as the measure of respiratory rate (breaths per minute).

The intercostal muscles on the other hand form two thin layers that run between the ribs and help shape and move the chest wall. The outer layer, the external intercostals is mainly active (burst activity in the trace) during inspiration to elevate the ribs, thus expanding the chest dimension, whereas, the inner layer, the internal intercostals compresses chest diameter, and is mainly involved in expiration (Hamberger, 1749). It is worth noting that this division is an oversimplification, and phase and level of activity also depends on dorsoventral location of the fibres and posture of the torso in both humans and animals (Butler et al., 2014). *In situ*, both muscle groups demonstrated predominant activity in phase with diaphragm activity (i.e., during inspiration) (Figure 1). Compared to inspiration, activity during expiration was modest in both intercostal groups, but proportionally to peak activity, the internal intercostals were generally more active during late expiration than external intercostals. However, both muscle sheets are very thin and closely apposed in juvenile rats, the perfusate is conductive, and electrode resistance low, therefore we cannot exclude some “bleeding” of electromyographic potential between muscle sheets.

To relate EMG activity to muscle rigidity, for each muscle type we determined the nadir, that is the minimum EMG amplitudes, during the diaphragm inter-burst interval (Figure 1B). This reflected both diaphragm and intercostal muscle activity during the expiratory phase. During the diaphragm burst phase, we determined the peak, that is the peak burst amplitude of the EMG signals (Figure 1B). This reflected both diaphragm and intercostal muscle activity during the inspiratory phase. An increase in the expiratory nadir and a decrease in the inspiratory peak EMG activity in the diaphragm and intercostal muscles would result in chest muscle rigidity (Campbell et al., 1995). Since either an increase in nadir or a decrease in peak EMG activity would translate into reduced tidal volume in an intact animal, we calculated the ratio of nadir to peak amplitude (nadir/peak) as a surrogate indicator of effect on tidal volume.

### 3.2 Comparison of the effects of fentanyl and morphine on respiratory muscle EMG activity

**FIGURE 2:**
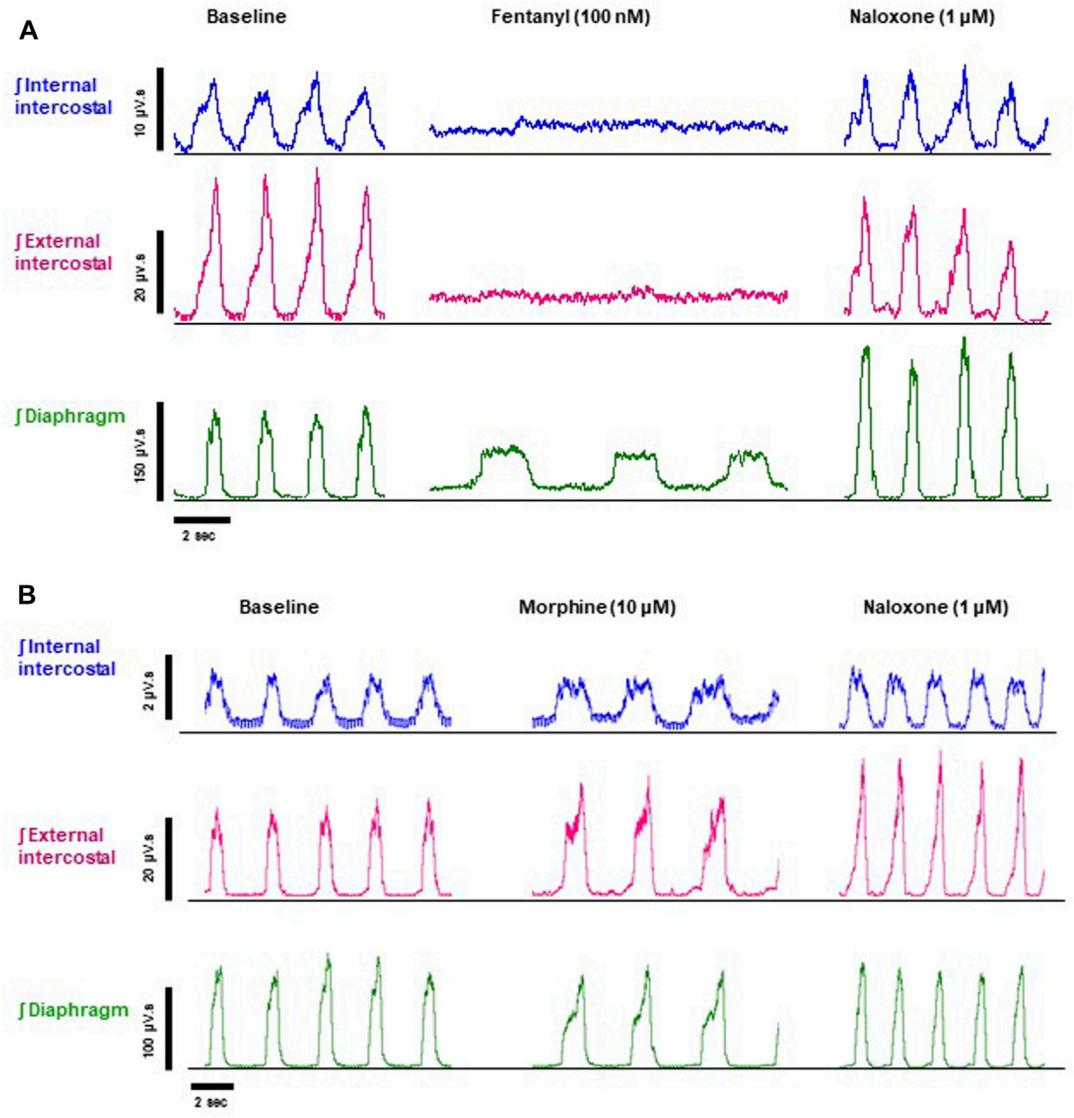
Effect of fentanyl and morphine on EMG activity in the diaphragm, external and internal intercostal muscles. **(A)** Baseline integrated EMG activity before drug addition (left panel), during perfusion with fentanyl (100 nM) (middle panel) and following addition of naloxone (1 μM) to the fentanyl-containing perfusate (right panel). **(B)** Baseline integrated EMG activity before drug addition (left panel), during perfusion with morphine (10 μM) (middle panel) and following addition of naloxone (1 μM) to the morphine-containing perfusate (right panel). Traces are typical of *n* = 5 experiments with each opioid agonist.

**FIGURE 3:**
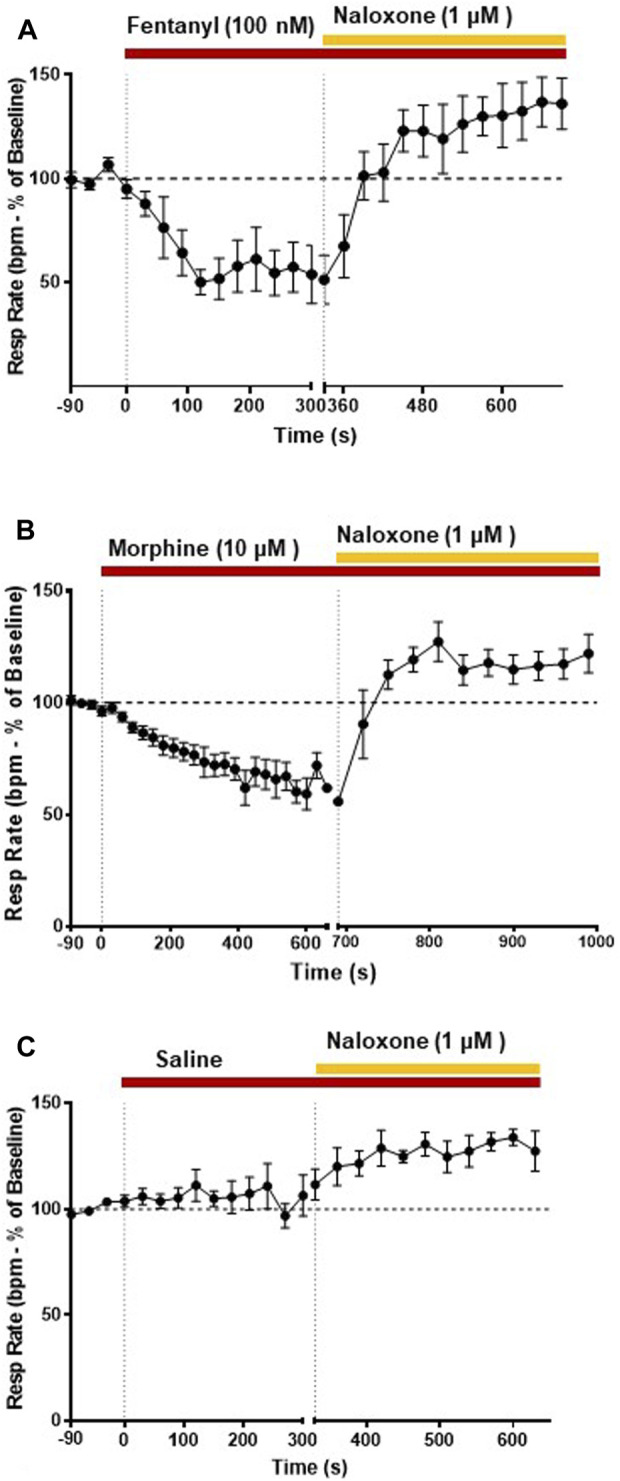
Effect of fentanyl and morphine on respiratory rate. Our main objective was to investigate the impact of various opioid agonists on muscle rigidity. For this, we specifically explored the concentrations of these agonists that resulted in a comparable decrease in respiratory rate, approximately around 40%. **(A)** Fentanyl (100 nM; *n* = 5) rapidly decreased respiratory rate reaching steady state depression within 2 min of administration. Naloxone (1 μM) rapidly reversed the fentanyl-induced respiratory depression. **(B)** Morphine (10 μM; *n* = 5) decreased respiratory rate with a slower rate of onset than fentanyl. Naloxone (1 μM) reversed the morphine-induced depression of respiratory rate. **(C)** Addition of saline did not alter respiratory rate. Data points were averaged in 30 s bins, and the subsequent change following drug administration calculated as a percentage of the pre-drug baseline. Error bars represent ±SEM. Statistical analyses of the effects of the overall opioid agonists are presented in the legend of Figure 8.

Our main objective was to investigate the impact of various opioid agonists on the electrical activity of respiratory muscles, specifically focusing on muscle rigidity. We started by examining opioids with well-established clinical or illicit use (i.e., fentanyl, morphine, carfentanil, and alfentanil) at concentrations that resulted in a comparable decrease in respiratory rate, approximately 40%. Addition of fentanyl (100 nM) to the perfusate rapidly depressed respiratory rate, reaching steady state bradypnea (−45% ± 1.5%, *n* = 5) 2 min after the start of drug administration (Figures 2A, 3A and Summary Figure 8A). Morphine (10 μM) also depressed respiratory rate but the effect was slower in onset, taking over 8 min to reach steady state (−36% ± 2%, *n* = 5) (Figures 2B, 3B and Summary Figure 8A). The slow rate of onset for morphine likely reflects its lower lipid solubility, which would slow distribution into the brain parenchyma (Pathan and Williams, 2012; Hill et al., 2019). Addition of vehicle (saline) (*n* = 5) to the perfusate in a separate group of preparations did not alter respiratory rate in equivalent timeframes (Figure 3C and Summary Figure 8A; statistical analysis of differences in respiratory depression between agonists is provided in Table 1). The bradypnea induced by fentanyl and morphine was rapidly reversed by addition of the opioid receptor antagonist, naloxone (1 μM), to the perfusate (Figures 2, 3A, B). Naloxone reversed the respiratory rate depression induced by fentanyl and morphine.

**TABLE 1:** Statistical analyses of differences between opioid agonists in depression of respiratory rate, shown in Figure 8A.

Tukey’s multiple comparisons (respiratory rate)	*p*-Value
Carfentanil vs. Fentanyl	<0.05
Carfentanil vs. Alfentanil	ns
Carfentanil vs. Norbuprenorphine	ns
Carfentanil vs. Morphine	ns
Carfentanil vs. Levorphanol	ns
Fentanyl vs. Alfentanil	ns
Fentanyl vs. Norbuprenorphine	ns
Fentanyl vs. Morphine	<0.05
Fentanyl vs. Levorphanol	ns
Alfentanil vs. Norbuprenorphine	Ns
Alfentanil vs. Morphine	ns
Alfentanil vs. Levorphanol	ns
Norbuprenorphine vs. Morphine	ns
Norbuprenorphine vs. Levorphanol	ns
Morphine vs. Levor	ns

**ANOVA table**	** **
DF	5
F (DFn, DFd)	F (5, 28) = 4.304
*p*-value	0.005

Abbreviations: degree of freedom (DF), F value (F), *p*-value (P).

**FIGURE 4:**
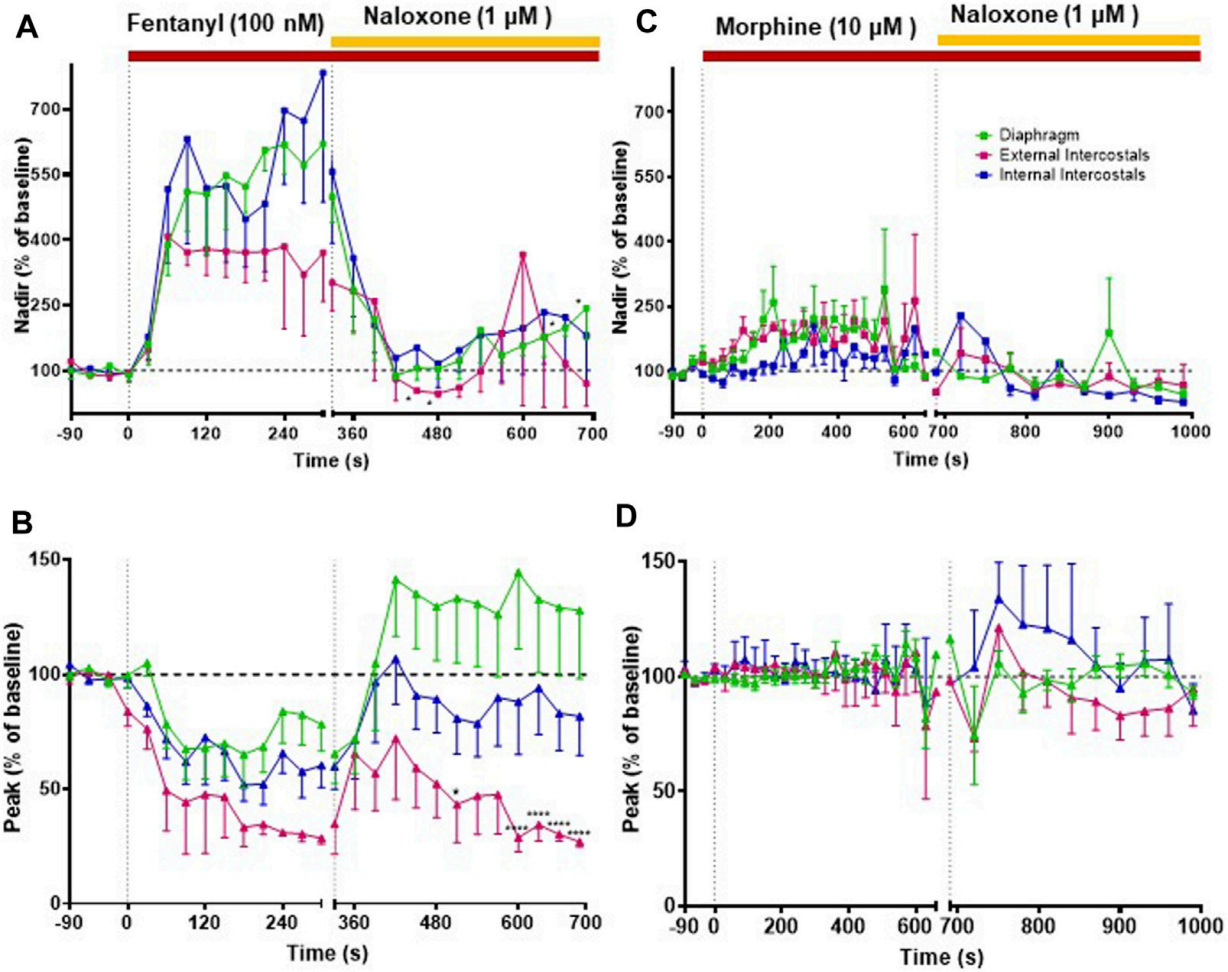
Effect of fentanyl and morphine on EMG activity in the diaphragm, external and internal intercostal muscles. **(A)** Fentanyl (100 nM; *n* = 5) produced a rapid increase in EMG activity at nadir in all three muscle types that was reversed by naloxone (1 μM), although the reversal by naloxone on the diaphragm decreased over time. **(B)** Fentanyl (100 nM) also decrease EMG activity at peak in all three muscle types. Naloxone (1 μM) reversed the fentanyl-induced peak depression in the diaphragm and the internal intercostals, but in the external intercostals the initial reversal decreased over time. **(C)** Morphine (10 μM) (*n* = 5) produced a small increase in EMG activity at nadir only in the diaphragm and external intercostals and not in the internal intercostals. **(D)** Morphine (10 μM) had no effect in EMG activity at peak in any of the muscle types. In all graphs the data points were averaged in 30 s bins, and the subsequent change following drug administration calculated as a percentage of the pre-drug baseline. Error bars represent ±SEM. Statistical analyses of the overall effects of the opioid agonists are presented in the legend of Figure 8. To indicate incomplete reversal by naloxone, *p < 0.05 and ****p < 0.0001 indicate significant difference from the pre-drug baseline as assessed by a two-way ANOVA with Šídák’s multiple comparisons test.

In addition to bradypnea, fentanyl (100 nM) also increased EMG activity at nadir and decreased peak activity in the diaphragm and, in particular, in the external and internal intercostal muscles, where the respiratory phase oscillation was abolished (Figures 2A, 4A, B and Summary Figures 8A-D; statistical analysis of differences in nadir and peak between agonists is provided in Table 2). Despite both morphine (10 μM) and fentanyl (100 nM) producing a similar respiratory rate depression (∼40%), their effects on EMG differed. Morphine did not have a significant effect on EMG activity in the internal intercostal muscle, and its impact on diaphragm and external intercostal EMG activity was only minimal at the nadir (lowest point) of respiratory activity. (Figures 2B, 4C, D). In contrast, fentanyl displayed notable effects on EMG activity in these muscles during the same conditions (see above). Addition of vehicle (saline) to the perfusate did not significantly alter EMG activity at nadir or peak in any of the respiratory muscles in the same timeframe (Summary Figures 8B, C). The addition of naloxone (1 μM) completely reversed the effect of fentanyl on EMG activity at nadir, although not maintained over time in the diaphragm and with fluctuations in external intercostal amplitude. The fentanyl-induced decrease in peak was reversed in the internal intercostals; in the diaphragm it was reversed but we also observed an increase above baseline; in the external intercostals, the partial rescue of peak amplitude declined over time (Figure 2A, 4A, B). The effects on EMG activity at nadir induced by morphine were fully reversed by naloxone (Figures 2B, 4C, D).

**TABLE 2:** Statistical analyses of differences in EMG peak and nadir amplitudes between opioid agonists, shown in Figure 8A.

Tukey’s multiple comparisons test	Diaphragm nadir *p*-value	Ext. Intercostal nadir *p*-value	Int. Intercostal nadir *p*-value	Diaphragm peak *p*-value	Ext. Intercostal peak *p*-value	Int. Intercostal peak *p*-value	Diaphragm Nadir/Peak *p*-value	Ext. Intercostal Nadir/Peak *p*-value	Int. Intercostal Nadir/Peak *p*-value
Carfentanil vs. Fentanil	<0.0001	ns	ns	<0.1	<0.0001	<0.0001	<0.0001	<0.0001	<0.01
Carfentanil vs. Alfentanil	<0.0001	ns	ns	ns	<0.0001	<0.0001	<0.0001	<0.0001	<0.01
Carfentanil vs. Norbuprenorphine	<0.0001	ns	ns	ns	<0.005	<0.01	<0.1	ns	ns
Carfentanil vs. Morphine	ns	<0.0001	= 0.0001	<0.1	ns	ns	ns	<0.01	<0.01
Carfentanil vs. Levorphanol	ns	<0.0001	<0.005	ns	ns	ns	ns	<0.01	<0.1
Fentanil vs. Alfentanil	ns	ns	ns	ns	<0.001	ns	ns	<0.001	ns
Fentanil vs. Norbuprenorphine	ns	ns	ns	0.01	<0.0001	<0.0001	ns	<0.0001	ns
Fentanil vs. Morphine	<0.0001	<0.0001	<0.0001	<0.0001	<0.0001	<0.0001	<0.0001	<0.0001	<0.0001
Fentanilt vs. Levorphanol	<0.0001	<0.0001	<0.001	<0.005	<0.0001	<0.0001	<0.0001	<0.0001	<0.0001
Alfentanilvs. Norbuprenorphine	<0.0001	ns	ns	ns	<0.0001	<0.0001	<0.001	<0.0001	ns
Alfentanil vs. Morphine	<0.0001	<0.0001	ns	= 0.0001	<0.0001	<0.0001	<0.0001	<0.0001	<0.0001
Alfentanil vs. Levorphanol	<0.0001	<0.0001	ns	ns	<0.0001	<0.0001	<0.0001	<0.0001	<0.0001
Norbuprenorphine vs. Morphine	<0.0001	<0.0001	<0.0001	0.01	ns	ns	<0.001	<0.0001	<0.0001
Norbuprenorphine vs. Levorphanol	<0.0001	<0.0001	<0.005	ns	ns	ns	<0.001	<0.0001	<0.001
Morphine vs. Levorphanol	ns	ns	ns	ns	ns	ns	ns	ns	ns

ANOVA table	Diaphragm Nadir	Ext. Intercostal Nadir	Int. Intercostal Nadir
DF	5	5	5
F (DFn,DFd)	F (5, 54) = 50.13	F (5, 56) = 21.10	F (5, 51) = 11.56
*p*-value	*p* < 0.0001	*p* < 0.0001	*p* < 0.0001

ANOVA table	Diaphragm Peak	Ext. Intercostal Peak	Int. Intercostal Peak
DF	5	5	5
F (DFn,DFd)	F (5, 41) = 9.821	F (5, 44) = 60.62	F (5, 46) = 41.85
*p*-value	*p* < 0.0001	*p* < 0.0001	*p* < 0.0001

ANOVA table	Diaphragm Nadir/Peak	Ext. Intercostal Nadir/Peak	Int. Intercostal Nadir/Peak
DF	5	5	5
F (DFn,DFd)	F (5, 56) = 25.95	F (5, 56) = 74.78	F (5, 52) = 21.93
*p*-value	*p* < 0.0001	*p* < 0.0001	*p* < 0.0001

Abbreviations: degree of freedom (DF), F value (F), *p*-value (P).

### 3.3 Effects of carfentanil and alfentanil on respiratory muscle EMG activity

**FIGURE 5:**
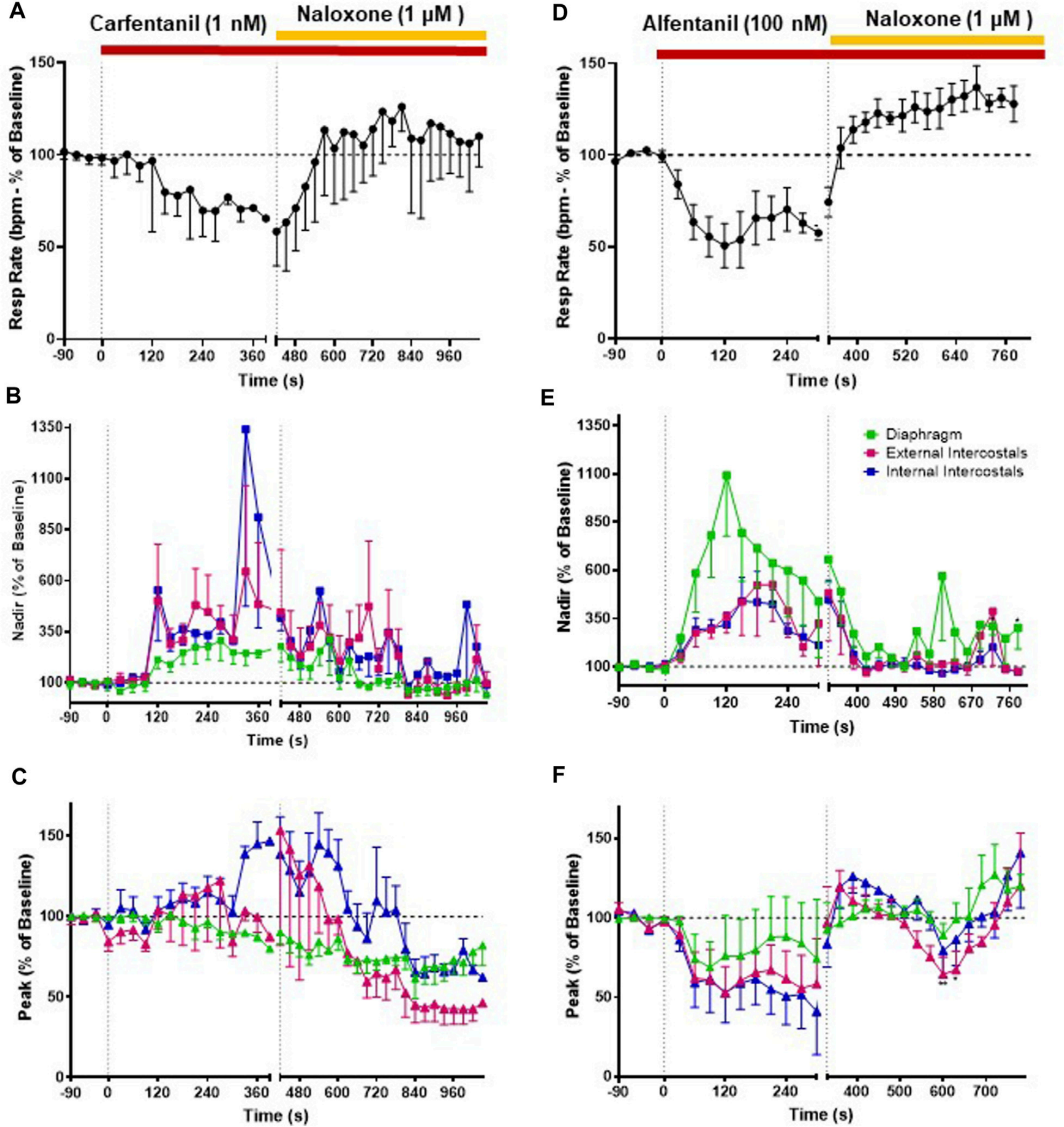
Effect of carfentanil and alfentanil on respiratory rate and EMG activity. **(A)** Carfentanil (1 nM; *n* = 5) depressed respiratory rate that was fully reversed by naloxone (1 μM). **(B)** Carfentanil (1 nM) increased nadir EMG activity in the internal and external intercostal muscles, with a smaller effect in the diaphragm; naloxone (1 μM) reversed the carfentanil-induced increase in nadir but with a slower rate of onset than that observed for reversal of fentanyl (see Figure 4). **(C)** Carfentanil (1 nM) had only a small effect on diaphragm peak EMG activity that was not reversed by naloxone and no effect on peak in the intercostal muscles. **(D)** Alfentanil (100 nM; *n* = 5) depressed respiratory rate, which was fully reversed by naloxone (1 μM). **(E)** Alfentanil (100 nM) increased EMG activity at nadir and decreased it at peak **(F)** in all three respiratory muscles. The alfentanil-induced increase in nadir in the external and internal intercostals was fully reversed by naloxone (1 μM); in the diaphragm the reversal of alfentanil-induced increase in nadir by naloxone was not fully maintained over time. The effect of alfentanil on EMG activity at peak was reversed by naloxone (1 μM) in all three respiratory muscles. Data points were averaged in 30 s bins, and the subsequent change following drug administration calculated as a percentage of the pre-drug baseline. Error bars represent ±SEM. Statistical analyses of the overall effects of the opioid agonists are presented in the legend of Figure 8. To indicate incomplete reversal by naloxone, *p < 0.05 and **p < 0.01 indicate significant differences from the pre-drug baseline data as assessed by a two-way ANOVA with Šídák’s multiple comparisons test.

Perfusion with carfentanil (1 nM) depressed respiratory rate (−34% ± 1% *n* = 5, Figure 5A and Summary Figure 8A; see Table 1 for statistical analysis) and increased nadir EMG activity in the internal and external intercostal muscles similarly to fentanyl (Figure 5B). However, carfentanil produced a smaller increase in nadir EMG activity in the diaphragm, a smaller decrease at peak EMG activity in diaphragm and no effect at peak in intercostal muscles (Figure 5C, Summary Figures 8B–G; Table 2 for statistical analysis), compared to the other high efficacy opioid agonists. To investigate if this difference was due to the low concentration used, we attempted to use a slightly higher concentration (1.5 nM). However, with carfentanil 1.5 nM (*n* = 3) there was a far greater reduction (85%) in respiratory rate than that observed with 1 nM the concentration that produced equivalent bradypnea (∼40%) to the other opioid agonists. Carfentanil (1.5 nM) did produce a significant decrease in peak EMG activity in all three muscles and the same increase in nadir as observed with 1 nM. Thus, carfentanil did produce respiratory muscle stiffness but only at a much higher level of bradypnea than fentanyl (Supplementary Figure S1).

Perfusion with alfentanil (100 nM) decreased respiratory rate (−40% ± 2% decrease, *n* = 5, Figure 5D), increased nadir and reduced peak EMG activity in all three respiratory muscles, similarly to fentanyl (Figures 5E, F; see Table 2 for statistical analysis of differences between agonists).

When naloxone (1 μM) was added to the perfusate, the effect on EMG activity at nadir induced by carfentanil (1 nM) was fully reversed, although its reversal by naloxone appears slower than that of the other opioid agonists. However, naloxone (1 μM) did not reverse the decrease in peak in the diaphragm, which rather continued to decrease. In the external and internal intercostals, we observed a decrease in peak in the presence of naloxone (1 μM) (Figures 5B, C). The alfentanil-induced increase in nadir was fully reversed by naloxone (1 μM) in the external and internal intercostals; in the diaphragm the reversal of alfentanil-induced increase in nadir by naloxone was not fully maintained over time (Figure 5E), whereas the effect on EMG activity at peak was reversed in all three respiratory muscles, although there have been fluctuations in the external intercostal amplitude (Figure 5F).

### 3.4 Comparison of *μ* opioid receptor ligands with different chemical properties and agonist efficacies at *μ* receptors

**FIGURE 6:**
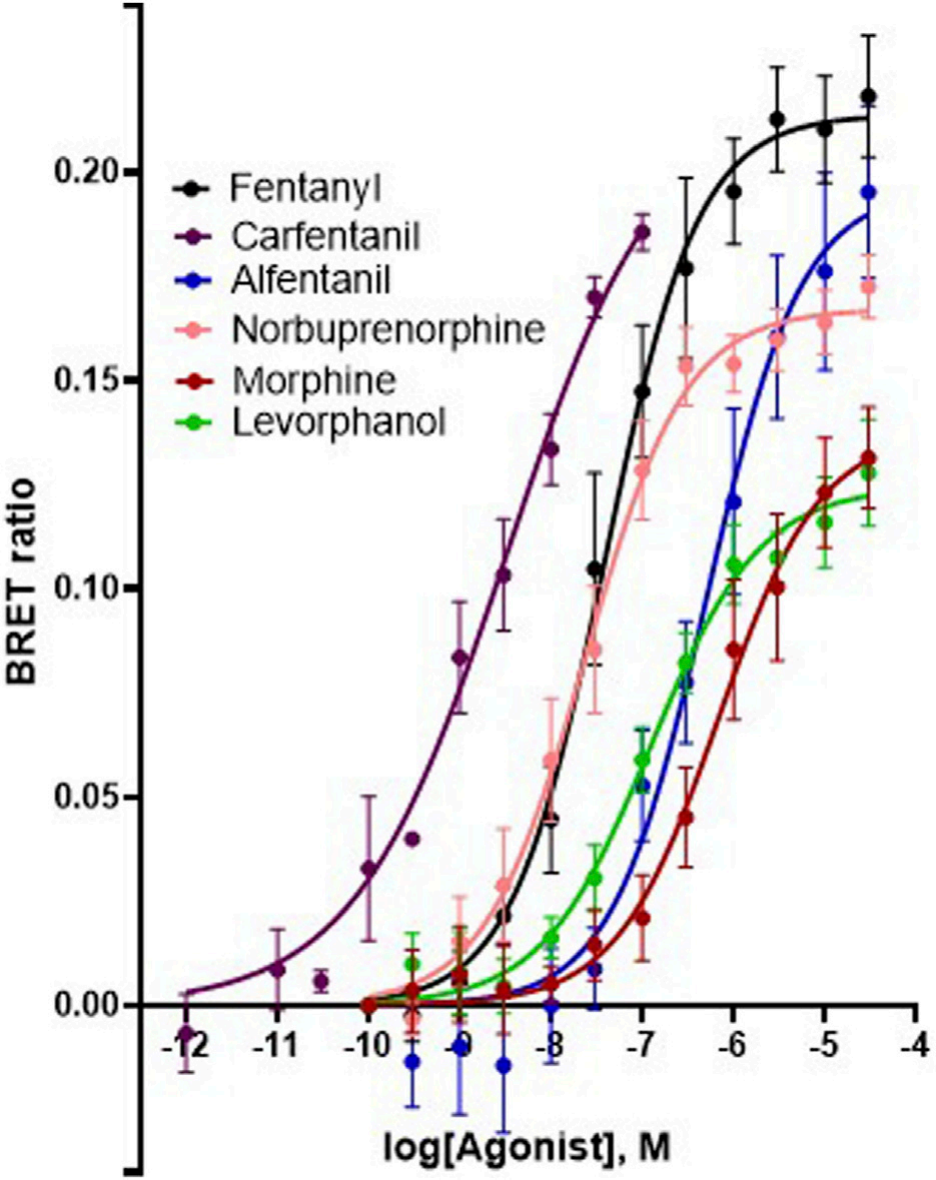
Concentration-response curves for opioid-induced G_i_ protein activation in HEK 293 cells expressing recombinant μ opioid receptors. G protein activation was measured as the decrease in BRET signal. Values of EC_50_ and maximum responses are recorded in Table 3. Data shown are means ± SEM, *n* = 5 for each drug.

In order to investigate what underlies the differential effects of the fentanyls and morphine on respiratory muscle EMG activity, we considered whether it might be related to differences in the chemical structure, lipid solubility or agonist intrinsic efficacy of the drugs. We therefore extended our study to include other non fentanyl opioid agonists, with different lipophilicity (see Table 3), and selected levorphanol and norbuprenorphine for comparison with the fentanyls and morphine. We determined the potency and agonist efficacy of all the opioid ligands to activate µ opioid receptors. We performed concentration-response studies of their ability to activate µ opioid receptors. To do this we used a BRET assay of G-protein activation in HEK-293 cells expressing rat µ opioid receptors (Figure 6). G protein coupled receptors that couple to Gi/Go proteins, such as the *μ* opioid receptor, have been extensively studied in HEK293 cells and behave as they do in single brain neurones with respect to relative potency and efficacy of opioid agonists (Ramos-Gonzalez et al., 2023). In a separate study we have examined the potency and efficacy of fentanyls on human *μ* opioid receptors expressed in AtT20 cells, a cell line with neuronal characteristics. There are no substantial differences from the results reported in the present manuscript (Alhosan, Kelly and Henderson, manuscript in preparation). Use of Emax does not allow discrimination between full agonists as they would all produce the same Emax but does discriminate between agonists that have lower maximum responses (Kelly et al., 2021). Based on the Emax values (Table 3; statistical analysis of differences between opioid agonist Emax is provided in Table 4) we can therefore categorise the relative efficacy of the drugs studied as carfentanil, fentanyl and alfentanil having “high” efficacy, norbuprenorphine, “moderate” efficacy, and morphine and levorphanol, “low” efficacy.

**TABLE 3:** Values of EC_50_, maximum response and lipid solubility of the tested opioid agonists.

Agonist	EC50 (nM) 95% CI	Maximum response (BRET ratio) 95% CI	Maximum response (% of fentanyl)	Lipid solubility (XLogP)*
Carfentanil 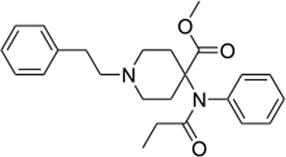	3 (1.1–49.4)	0.2 (0.17–0.35)	102	3.8
Fentanyl 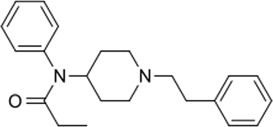	39 (25–62)	0.2 (0.19–0.232)	100	4
Alfentanil 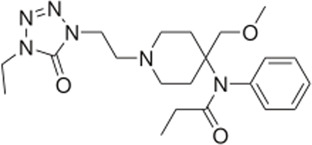	508 (250–1,400)	0.196 (0.16–0.24)	92	2.2
Norbuprenorphine 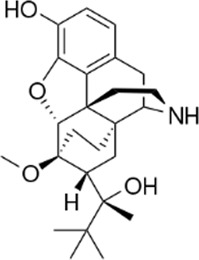	24 (15–37)	0.167 (0.15–0.17)	78	3.8
Morphine 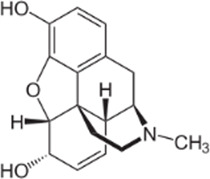	437 (217–1,450)	0.138 (0.11–0.19)	65	0.49
Levorphanol 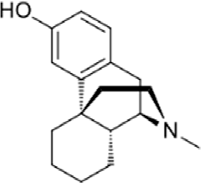	124 (71–260)	0.125 (0.11–0.14)	58	3.29

EC_50_ and maximum response data were derived from the concentration-response curves in Figure 6 (*n* = 5 for each agonist). Numbers in parentheses indicate 95% confidence intervals.

^a^ Data from PubChem.

**TABLE 4:** Statistical analyses of differences between opioid agonists in EC_50_ and aximum response for G protein activation.

Tukey’s multiple comparison	*p*-Value summary (EC50)	*p*-Value summary (emax)
Morphine vs. Levorphanol	ns	Ns
Morphine vs. Norbuprenorphine	<0.001	<0.01
Morphine vs. Carfentanil	<0.0001	<0.05
Morphine vs. Fentanyl	<0.05	<0.001
Morphine vs. Alfentanil	ns	<0.01
Levorphanol vs. Norbuprenorphine	ns	<0.001
Levorphanol vs. Carfentanil	<0.0001	= 0.005
Levorphanol vs. Fentanyl	ns	<0.0001
Levorphanol vs. Alfentanil	ns	<0.001
Norbuprenorphine vs. Carfentanil	<0.05	ns
Norbuprenorphine vs. Fentanyl	ns	= 0.0005
Norbuprenorphine vs. Alfentanil	<0.001	ns
Carfentanil vs. Fentanyl	<0.01	ns
Carfentanil vs. Alfentanil	<0.0001	ns
Fentanyl vs. Alfentanil	<0.05	ns

ANOVA table	
DF	5
F (DFn, DFd)	F (5, 34) = 18.38
*p*-value	*p* < 0.0001

Abbreviations: degree of freedom (DF), F value (F), *p*-value (P) and *p*-value summary.

### 3.5 Effects of norbuprenorphine and levorphanol on respiratory muscle EMG activity

**FIGURE 7:**
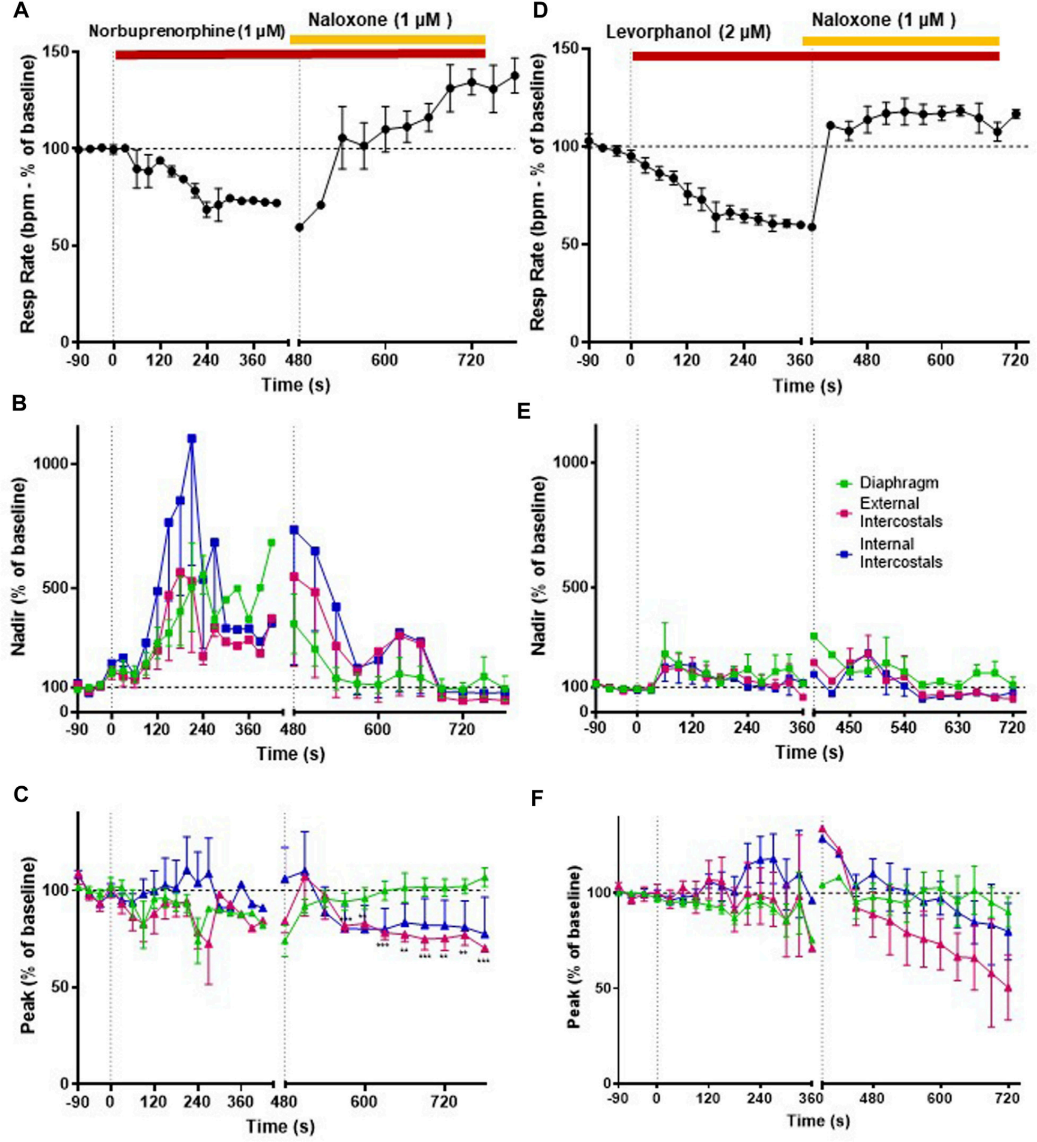
Effect of norbuprenorphine and levorphanol on respiratory rate and EMG activity. **(A)** Norbuprenorphine (1 μM; *n* = 5) depressed respiratory rate that was fully reversed by naloxone (1 μM). **(B)** Norbuprenorphine (1 μM) significantly increased nadir EMG amplitude in all three respiratory muscles, that was reversed by naloxone (1 μM). **(C)** Norbuprenorphine (1 μM) produced only a small decrease in peak EMG amplitude in diaphragm and external intercostals but had no effect on peak EMG amplitude in internal intercostals. Naloxone (1 μM) reversed the decrease in peak in the diaphragm but not in the external intercostals. **(D)** Levorphanol (2 μM; *n* = 5) depressed respiratory rate, which was fully reversed by naloxone (1 μM). **(E)** Levorphanol (2 μM) only initially produced a small increase in nadir EMG activity in the respiratory muscles, but this effect was not maintained. **(F)** Levorphanol (2 μM) did not produce any effect at the peak EMG amplitude. Data points were averaged in 30 s bins, and the subsequent change following drug administration calculated as a percentage of the pre-drug baseline. Error bars represent ±SEM. Statistical analyses of the overall effects of the opioid agonists are presented in the legend to Figure 8. To indicate incomplete reversal by naloxone **p < 0.01 and ***p < 0.001 denote significant differences from the pre-drug baseline data as assessed by a two-way ANOVA with Šídák’s multiple comparisons test.

**FIGURE 8:**
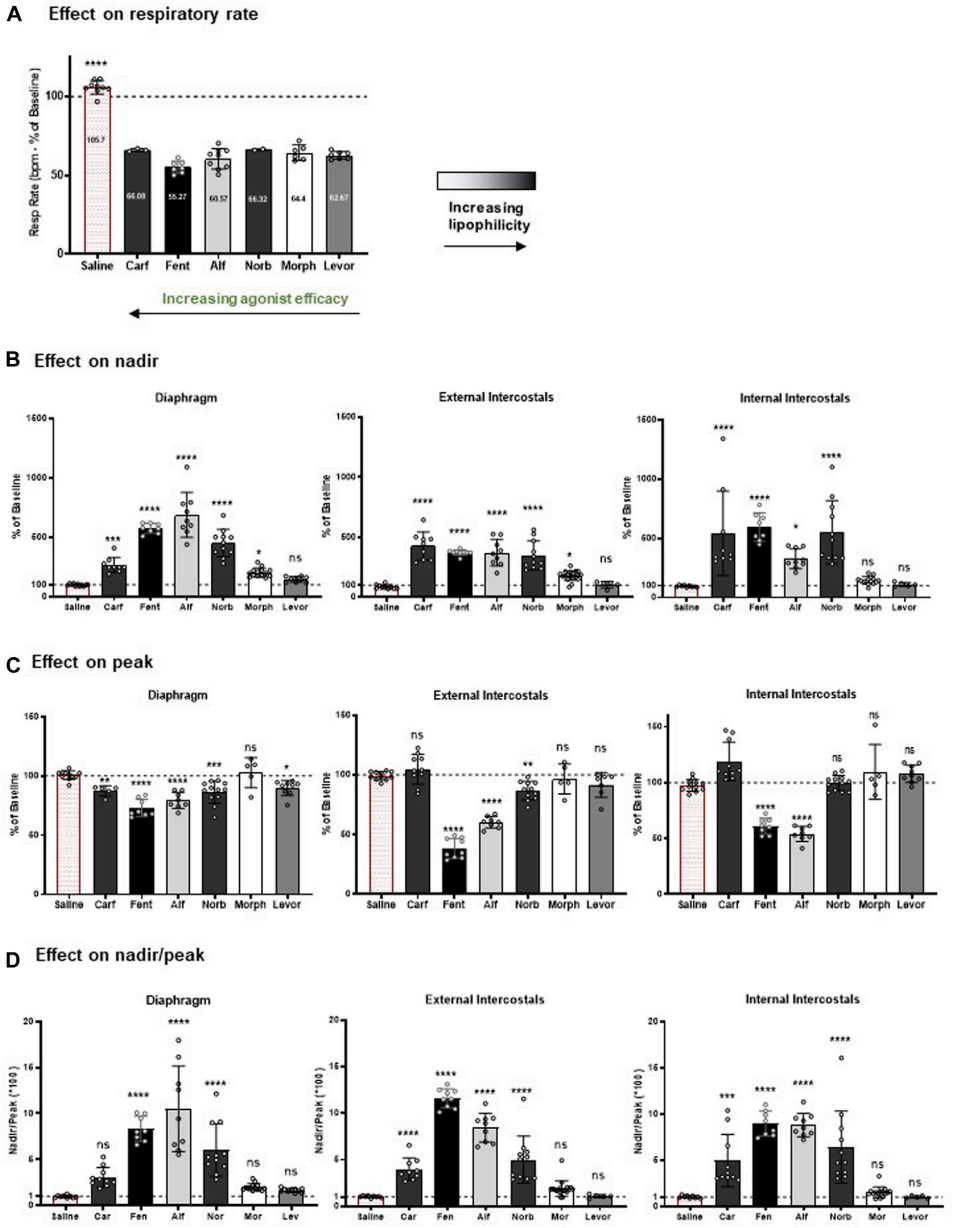
Summary of the effects of the opioid agonists on respiratory rate and EMG amplitude. **(A)** At the concentration tested (1 nM carfentanil, 100 nM fentanyl, 100 nM alfentanil, 1 μM norbuprenorphine, 10 μM morphine and 2 μM levorphanol), each opioid agonist decreased respiratory rate by approximately 40%, with no statistically significant difference between the degree of effect produced by the agonists. **(B)** Effect on EMG nadir in diaphragm, external intercostal muscles and internal intercostal muscles. **(C)** Effect on EMG peak in diaphragm, external intercostal muscles and internal intercostal muscles. **(D)** Effect on nadir/peak ratio in diaphragm, external intercostal muscles and internal intercostal muscles. For each parameter, data are presented as mean ± SD (*n* = 5) of steady state data points. *** and **** indicate significant difference from the vehicle group (saline) at *p* < 0.001 and *p* < 0.0001 respectively as assessed by one-way ANOVA with Dunnett’s multiple comparisons test; ns - not significant. Evaluation of the results of ANOVA comparisons are shown in Table 5.

We then went on to determine the effect of norbuprenorphine and levorphanol on respiratory muscle EMG activity in the *in situ* arterially perfused working heart–brainstem preparation. We used a concentration of each agonist that produced equivalent bradypnea, −32.7% and −37.4% respectively (Figures 7A, D, 8A; statistical analysis of differences in respiratory depression between agonists is provided in Table 1) that persisted for the duration of agonist application. Norbuprenorphine (1 μM), increased nadir EMG activity in all three respiratory muscles, similarly to fentanyl (Figure 7B and Summary Figure 8B). It produced a smaller reduction than fentanyl in peak EMG activity in the diaphragm and in the external intercostal muscles (Figure 7C and Summary Figure 8C). Levorphanol (2 μM) did not produce a sustained increase in nadir EMG activity in any of the respiratory muscles (Figure 7E and Summary Figure 8B; statistical analysis is provided in Table 2). Levorphanol also did not affect peak EMG activity in any of the respiratory muscles, similarly to morphine (Figure 7F and Summary Figure 8C). The effects of norbuprenorphine and levorphanol on depression of respiratory rate were reversed on addition of naloxone (1 μM) to the perfusate (Figures 7A, D). The effects of norbuprenorphine on EMG activity at nadir were reversed by naloxone (1 μM) in all three muscles, even though more slowly than other opioid agonists. The effects on EMG activity at peak were reversed in the diaphragm but in the external and internal intercostal muscles, we observed a further reduction in the peak amplitude on addition of naloxone (Figures 7B, C).

**TABLE 5:** ANOVA Evaluation of the graphs shown in Figure 8.

Respiratory rate	Graph 8A		
DF	6		
F	119.4		
*p*-value	<0.0001		

Nadir	Graph 8B_1_	Graph 8B_2_	Graph 8B_3_
DF	6	6	6
F	64.64	30.69	14.3
*p*-value	<0.0001	<0.0001	<0.0001

Peak	Graph 8C_1_	Graph 8C_2_	Graph 8C_3_
DF	6	6	6
F	15.83	68.15	40.97
*p*-value	<0.0001	<0.0001	<0.0001

Nadir/Peak	Graph 8D_1_	Graph 8D_2_	Graph 8D_3_
DF	6	6	6
F	29.29	82.89	26.28
*p*-value	<0.0001	<0.0001	<0.0001

Abbreviations: degree of freedom (DF), F value (F), *p*-value (P).

Comparing the effects of all drugs on respiratory muscle EMG activity with chemical structure, lipophilicity and agonist efficacy, our results indicate that muscle rigidity can be produced both by fentanyls and the non fentanyl opioid agonist norbuprenorphine. There is a good correlation between the ability of opioids to induce muscle rigidity and their agonist efficacy. High lipophilicity alone (the case with levorphanol) is not enough to produce muscle rigidity.

Figure 8D shows the effect of the opioid agonists on the ratio of nadir to peak in each of the three muscle groups. The higher efficacy opioid agonists, alfentanil, fentanyl and norbuprenorphine, increased the ratio of nadir to peak in all three muscles, whereas carfentanil increased the ratio of nadir to peak in the intercostal muscles but not the diaphragm. In contrast, the low efficacy agonists, morphine and levorphanol, did not increase the ratio of nadir to peak in any of the three respiratory muscles. These data indicate that, in general, at a dose that produces ∼40% depression of respiratory rate, the high efficacy agonists would reduce tidal volume in intact animals, whereas low efficacy agonists would not. (Statistical analysis of differences in nadir/peak between agonists is provided in Table 2).

Respiratory arrest was only observed at the highest tested concentration of carfentanil. Importantly, none of the *μ* opioid agonists examined in our study induced intermittent apnoeas, characterized by temporary cessations in breathing.

### 3.6 Effect of fentanyl on upper airway resistance

**FIGURE 9:**
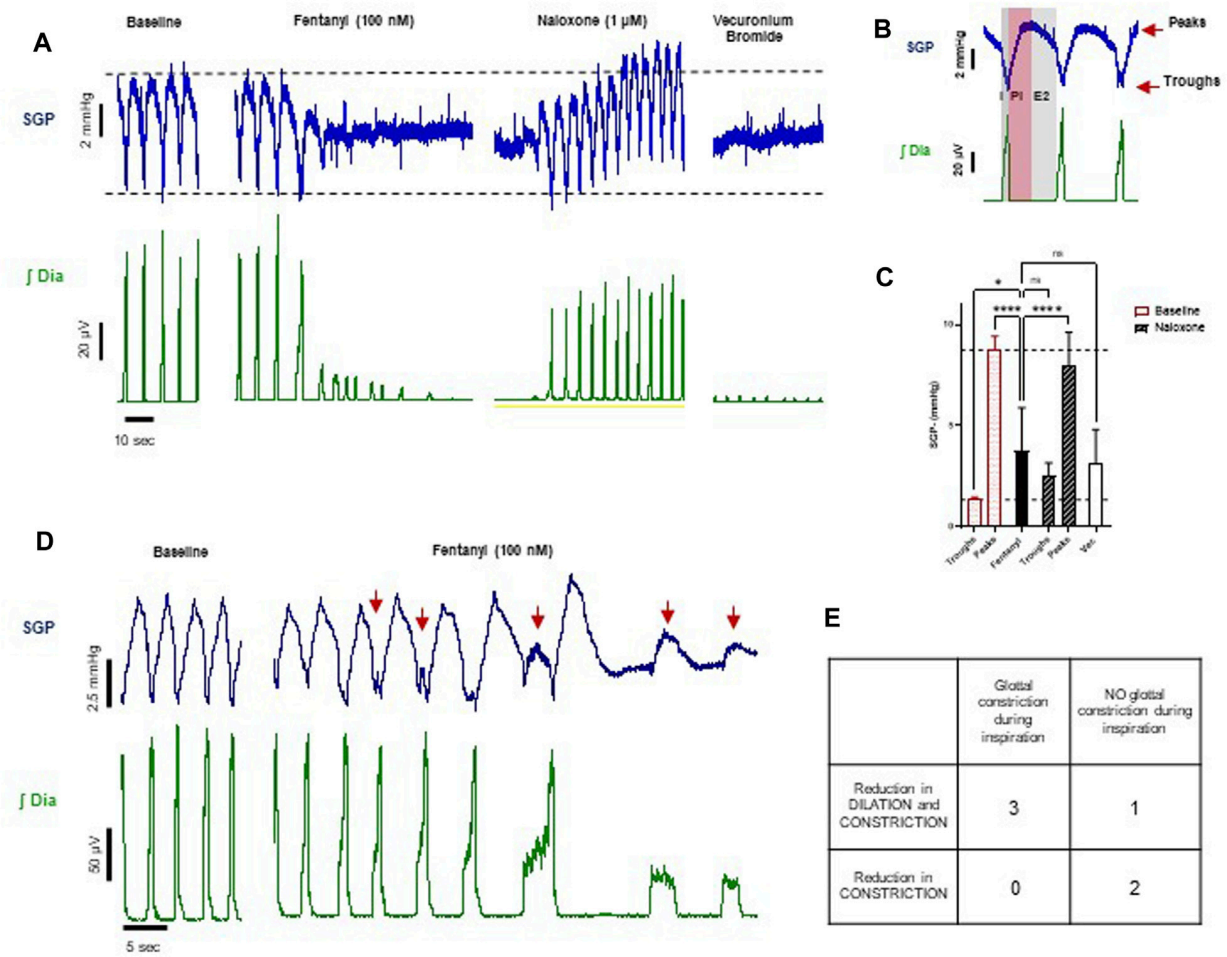
Effect of fentanyl on subglottal pressure (*n* = 6). **(A)** Traces from an experiment representative of the three experiments showing changes in both dilation and constriction of the glottis in response to drugs. Baseline SGP before drug addition (first panel), phasic SGP pressure waves waning towards a mean pressure level in the presence of fentanyl (100 nM) (second panel), SGP after subsequent addition of naloxone (1 μM) (third panel) and finally SGP after addition of vecuronium bromide (fourth panel). **(B)** Expanded timescale of respiratory cycle showing the typical subglottal pressure (SGP) recording from the larynx (blue trace) and diaphragm EMG (green trace), showing the three-phasic breathing pattern of the respiratory cycle. This comprises inspiration (I; dark grey area), post inspiration (PI; pink area), and late expiration (E2; light grey area). The top red arrow indicates the peak and the bottom red arrow indicates the trough of the subglottal pressure trace. **(D)** Traces from an experiment representative of the three experiments with glottal constriction during the inspiratory phase, as indicated by the red arrows. **(C)** Quantitative analysis of the effect of fentanyl (100 nM), naloxone (1 μM) and vecuronium bromide on, on peaks and troughs of SPG. For fentanyl and vecuronium bromide, SGP is calculated as average of values at the steady state. Data are presented as mean ± SD. ****p < 0.0001 and *p < 0.05 as assessed by one-way ANOVA with Šídák’s multiple comparisons test. **(E)** Table summarising the experiments based on the visual inspection of the traces.

In the arterially perfused working heart-brainstem preparation, subglottal pressure (SGP) recordings from the larynx under control conditions displayed the typical three-phasic pattern of the respiratory cycle (Figure 9B): during inspiration (I, coincident with diaphragm activity), SGP decreased reflecting laryngeal dilation inferred from the decrease in airway resistance which results from abductor muscle contraction; in the subsequent post-inspiratory phase (PI), also known as passive expiration, SGP increased reflecting laryngeal constriction inferred from the increase in airway resistance which results from adductor muscle contraction. Laryngeal dilation is thought to reduce expiratory airflow and counteract the intrinsic recoil of the expanded lungs (Dutschmann and Dick, 2012). In the later expiration (E2), also known as active expiration, SGP values reached a steady state, thought to maintain volume and prevent collapse of the lower airways.

Fentanyl (100 nM, *n* = 6) did not produce the expected increase in SGP that has been described in the literature (Willette et al., 1982; Scamman, 1983; Fahnenstich et al., 2000; Miner et al., 2021). Instead, we observed that, at steady state, SGP peaks decreased on average by more than 50% and troughs increased by almost three-fold, trending towards a mean SGP level (Figures 9A, C), which does not reflect an overall increase in laryngeal resistance. This suggests that fentanyl induced a reduction in both glottal dilation and constriction (abductor and adductor muscle activity, respectively) and therefore a loss in the larynx function of maintaining airway patency and regulating airflow. This effect was reversed by addition of naloxone (1 μM), although the trough values were slightly elevated. The effect of the neuromuscular blocker, vecuronium bromide, on SGP peaks and troughs did not differ significantly from the effects of fentanyl (Figures 9A, C). This confirms that, *in situ*, fentanyl might cause full relaxation of the laryngeal muscles.

We also observed in three out of six experiments that after fentanyl addition but prior to the drug effect reaching steady state, there were transient increases in SGP during inspiration (i.e., out of phase) (Figures 9D, E). These events occurred in some respiratory cycles and varied in amplitude (see the first three arrows in Figure 9D, compared to the last two arrows). In one experiment, SGP retained phasic activity during steady state, but with a reduced laryngeal dilation during the inspiratory phase. In the remaining two experiments, fentanyl abolished the post-inspiratory laryngeal constriction. This indicates that in the *in situ* arterially perfused preparation fentanyl may affect different states of larynx function, possibly depending on the different laryngeal muscles involved when fentanyl distributes into the brainstem. Although our experiments did not show an average increase in airflow resistance, the larynx lost its ability to maintain airway patency during inhalation and regulate airflow during exhalation.

## 4 Discussion

The objective of this study was to examine the potential factors that could contribute towards the ability of fentanyls to produce respiratory muscle rigidity which, along with their potency to induce bradypnea and hypoxia, makes these drugs highly lethal in overdose. The factors we considered to understand the differences in the effect on EMG activity between the opioids were (i) the chemical nature of the drugs (to determine whether muscle rigidity is a property of only fentanyl and its analogues or whether structurally unrelated opioid agonists can also induce this phenomenon), (ii) ligand lipid solubility (which may influence access to specific brain regions involved in generating muscle rigidity) and (iii) agonist intrinsic efficacy at *μ* opioid receptors (to examine whether only high efficacy agonists can induce the response). We used the arterially perfused working heart-brainstem preparation of the rat which allowed us to infuse drugs for prolonged periods at known concentrations whilst recording spontaneous respiratory rate and the electrical activity of respiratory muscles. We observed that, when applied at concentrations that produced approximately the same degree of bradypnea (equipotent concentrations), fentanyl, alfentanil and carfentanil as well as the non-fentanyl opioid, norbuprenorphine, increased expiratory nadir in all three respiratory muscles although the effect of carfentanil on the diaphragm, but not the intercostals, was less than that of the other fentanyls. These opioid agonists all have relatively high intrinsic agonist efficacy and high lipophilicity but norbuprenorphine is structurally dissimilar to the fentanyls being a thebaine derivative (Table 3). In contrast, levorphanol, a morphinan of lower agonist efficacy but high lipophilicity, and morphine, the prototypic opioid which has both lower agonist efficacy and lower lipophilicity, produced little or no change in expiratory nadir at the concentration tested. These observations suggest that the most important factor underlying opioid-induced changes in respiratory muscle electrical activity is agonist intrinsic efficacy although high lipophilicity may also be important in enabling such drugs to access the brain site(s) at which this effect is produced. It will be of interest to determine the effects of other high efficacy opioid agonists that are drugs of abuse such as the nitazenes on respiratory muscle activity (Rackam, 1980).

A possible explanation for why only high efficacy *μ* opioid agonists are able to induce respiratory muscle rigidity would be that brain regions involved in initiating muscle rigidity may express a reduced level of *μ* opioid receptors. In situations of low receptor expression then the concentration-response curve for lower efficacy (partial) agonists will be flattened whereas for high efficacy (full) agonists it will only be shifted to the right (Kelly, 2013). Low levels of receptor expression would therefore reduce or even abolish the response to lower efficacy agonists. This phenomenon has previously been demonstrated in the rat vas deferens where high efficacy *μ* opioid agonists inhibit neurotransmission but morphine, a lower efficacy agonist, does not, but acts as an antagonist (Sheehan et al., 1988). However, when we examined the ability of morphine to antagonise fentanyl in the rat perfused working heart-brainstem preparation, we did not observe antagonism (data not shown). This may be due to morphine being unable to access all the receptor populations that fentanyl accesses.

Adequate ventilation of the lungs requires coordinated movements of the diaphragm and the chest wall (Sterling, 1979). Augmented chest wall muscle tone reduces chest wall compliance, impairing ventilation (Coruh et al., 2013). In our experiments, the increase in expiratory nadir and the decrease in the inspiratory peak EMG activity observed in all three respiratory muscles resulted in reduced/loss of phasic activity, which is consistent with chest wall rigidity (Campbell et al., 1995). The increase in expiratory EMG activity, passive at rest (Mantilla et al., 2013; Aliverti, 2016), reflects reduced ability of inspiratory muscles to relax properly during expiration and therefore increased muscle tone of the chest wall. On the other hand, the decrease in peak EMG amplitude would predict a reduced tidal volume in an intact animal. For this reason, we calculated the ratio of nadir to peak amplitude (nadir/peak) as a surrogate indicator of effect on tidal volume, as an increase in nadir along with a decrease in peak EMG activity would result in reduced tidal volume.

Our data indicate that, high efficacy opioid agonists would likely reduce tidal volume in intact animals, whereas low efficacy agonists would not. These findings are in line with our previous plethysmography experiments made in freely moving mice, showing that fentanyl depressed both respiratory rate and tidal volume, whereas morphine only at the highest dose had a small effect on tidal volume (Hill et al., 2019). In paralysed preparations fentanyl did decrease the amplitude of phrenic motor output, implying that reductions in tidal volume induced by fentanyl are governed by significant central mechanisms (Saunders and Levitt, 2020). In our experiments carfentanil produced a smaller effect on diaphragm activity compared to fentanyl, alfentanil and norpbuprenorphine. Preliminary data in freely moving mice suggest that carfentanil does not produce a marked decrease in tidal volume at doses which depress respiratory rate (Cavallo, Abdala and Henderson, unpublished observations) which correlates with the reduced ability of carfentanil to induce respiratory muscle rigidity in this study. We have recently reported that carfentanil shows significant bias in favour of arrestin recruitment over G protein signalling (Ramos-Gonzalez et al., 2023). How this could affect its ability to induce respiratory muscle rigidity is not clear; enhanced *μ* opioid receptor desensitization is unlikely as, at the concentration studied in the plethysmography experiments, bradypnea was maintained throughout the period of carfentanil exposure. It is important to note that one key limitation of our study was not conducting a full concentration-response analysis for each agonist *in situ*. However, our choice of experimental design stemmed from a practical constraint related to ethical considerations. Due to the potential rapid development of tolerance, only a single dose of opioid receptor agonist could be used in each preparation. The extensive number of animals that would be required to perform comprehensive concentration-response curves for each agonist was substantial and adhering to the principles of the 3Rs (Replacement, Reduction, and Refinement) was paramount in our experimental design.

While we recognize the significance of dose-response analysis in precisely comparing various opioid receptor agonists, the ethical and logistical challenges associated with the large number of animals involved necessitated the approach we undertook. By implementing statistical analyses and careful experimental design, we aimed to maximize the information obtained from the available data while minimizing the number of animals used.

The mechanisms underlying opioid-induced muscle rigidity, first described by Hamilton and Cullen in 1953 as “a pronounced degree of rigidity and increased muscle tone”, remain poorly understood. This rigidity decreases chest wall compliance (Blasco et al., 1986), and abdominal musculature, resulting in the “wooden chest syndrome” (Coruh et al., 2013). Several nuclei have been identified to be involved in the skeletal muscle rigidity: the nucleus raphe pontis (NRP) within the reticular formation (Blasco et al., 1986) and the locus coeruleus through the activation of the coerulospinal noradrenergic pathway following *μ* receptor activation: this process may involve a subsequent indirect excitatory action through α_1_-, and an inhibitory action through α_2_ adrenoceptors, in the spinal cord (Jerussi et al., 1987; Lui et al., 1989; Tsou et al., 1989). The α_2_ agonist dexmedetomidine used to reverse skeletal muscle rigidity also reversed diaphragm muscle rigidity (Campbell et al., 1995; Haouzi and Tubbs, 2022), suggesting that both peripheral and respiratory muscle rigidity may involve an adrenergic component.

In each experiment, we introduced vecuronium bromide, a neuromuscular blocker, at the conclusion of the procedure. This addition served three primary purposes. Firstly, it allowed us to establish the baseline noise level in each electrode. Secondly, it confirmed if the observed agonist-mediated muscle effects remained unaffected by factors downstream of the neuromuscular junction. Thirdly, in the case of subglottal pressure experiments, vecuronium bromide was employed to ascertain the residual level of subglottal pressure in instances where laryngeal muscle activity was fully relaxed, thereby enabling a precise assessment of glottal function in the presence of *μ* opioid receptor agonists compared its resting state. Vecuronium bromide ceased EMG activity, to the same remaining baseline noise level when the preparation was euthanized by cessation of perfusion. This provides compelling and conclusive evidence that the observed agonist-mediated muscle rigidity was not influenced by factors downstream of the neuromuscular junction.

In our *in situ* heart–brainstem preparation the bradypnea induced by all opioid agonists was reversed by naloxone.

Fentanyl and its analogues are known to cause reflex excitation of the recurrent laryngeal nerve (RLN) correlated with a large increase in the laryngeal resistance to airflow (Willette et al., 1982) and closure of the glottis (vocal cords) and supraglottic structures (Abrams et al., 1996; Bennet et al., 1997; Miner et al., 2021). The upper airway muscles play an important role in ventilation, by determining the upper airway patency and dynamically adapting the airway resistance to airflow (Bautista and Dutschmann, 2014). At steady state, in the presence of fentanyl, we observed that phasic SPG pressure waves waned towards a mean pressure level. This suggests a reduction in both glottal dilation and constriction (abductor and adductor muscle activity, respectively). Indeed, on average, the effect of fentanyl did not significantly differ from that of neuromuscular blockade which produces full relaxation of the laryngeal muscles. The effect of fentanyl was substantially reversed by naloxone. Our finding is at odds with previous studies that described laryngeal closure in response to fentanyl (Willette et al., 1982; Scamman, 1983; Fahnenstich et al., 2000; Miner et al., 2021). We do not have a definitive explanation for this difference, but it may relate to differences between the *in vivo* situation and the *in situ* arterially perfused working heart-brainstem preparation.

The *in situ* preparation has an intact respiratory circuitry that largely produces an “*in vivo*-like” 3 phase respiratory motor output pattern in the absence of anaesthesia, but it cannot be fully equated to eupnea *in vivo*. A main difference is the significantly lower breathing frequency compared with *in vivo*. As the lungs have been removed, there is no phasic lung inflation and pulmonary stretch receptor feedback, and there is diminished chemosensory reflex due to hyperoxia and pH maintenance by the perfusion solution (Dutschmann and Paton, 2005; Saunders and Levitt, 2020). In our experiments, we observed synchronized inspiration-related activity in both the internal and external intercostal muscles, because both muscle sheets are very thin and closely positioned in juvenile rats, and due to the conductive nature of the perfusate and low electrode resistance we cannot exclude the possibility of some overlap in electromyographic signals between the muscle sheets; additionally, whether these muscles provide an advantage for inhalation or exhalation can change depending on factors such as the species, body posture, and the demand for breathing. For instance, while the external intercostals show minimal activity during quiet breathing, their importance increases during forced breathing (Rimmer et al., 1995; De Troyer et al., 2005; Butler et al., 2014). Furthermore, SGP recordings cannot be related to activities of defined muscle groups of the larynx and have to be seen as measurement of overall glottal resistance during a respiratory cycle (Dutschmann and Paton, 2005).

It is worth noting that we have observed inter-individual variability in responses to *μ* opioid receptor agonists, particularly in the case of intercostal muscle nadir and subglottic pressures. Interestingly, we found relatively little inter-individual variation in respiratory rate suppression by the same drugs. Importantly, marked intra- and/or inter-individual variability has been reported in human studies, encompassing both the euphoric effects of opioids such as fentanyl (Hoehe, 2007), as well as their impact on respiratory function, as seen with heroin (Tas et al., 2020). These findings align with existing evidence that highlights the inconsistent relationship between opioid dosage and the risk of overdose.

## 5 Conclusion

In this study we have shown how opioid agonists differentially affect skeletal respiratory muscles in the *in situ* rat working heart–brainstem preparation. Our results suggest that the ability of opioids to induce respiratory muscle rigidity is not a property of only fentanyl derivatives, as also the non-fentanyl opioid norbuprenorphine was able to affect the electrical activity of respiratory muscles; nor a property of high lipophilicity, as levorphanol was not able to affect the electrical activity of respiratory muscles. Rather, the ability to cause muscle rigidity correlates with opioid agonist intrinsic efficacy. Unlike previous, studies, we found that fentanyl did not produce an overall increase in laryngeal airway resistance. Nevertheless, in our model, the larynx lost its ability to maintain airway patency and regulate airflow, which is likely to contribute to the ventilatory collapse caused by fentanyl *in vivo*.

## Data Availability

The raw data supporting the conclusions of this article will be made available by the authors, without undue reservation.

## Supplementary material

Supplementary Figure S1 Effect of 1.5 nM carfentanil (n = 3) on respiratory rate and on EMG activity in the diaphragm, external and internal intercostal muscles. **(A)** Baseline integrated and raw EMG recordings before drug addition (left panel), during perfusion with carfentanil (1.5 nM) (middle panel) and following addition of naloxone (1 μM) to the carfentanil-containing perfusate (right panel). **(B)** Carfentanil (1.5 nM) depressed respiratory rate by 85%, that was fully reversed by naloxone (1 μM). **(C)** Carfentanil (1.5 nM) increased nadir EMG activity in the internal and external intercostal muscles, with a smaller effect in the diaphragm; naloxone (1 μM) didn’t fully reverse carfentanil-induced increase of nadir. **(D)** Carfentanil (1.5 nM) decreased peak EMG activity in all the respiratory muscles; naloxone (1 μM) reversed the carfentanil-induced decrease in peak but with a slower rate of onset than that observed for reversal of fentanyl (see Figure 4). Data points were averaged in 30 second bins, and the subsequent change following drug administration calculated as a percentage of the pre-drug baseline. Error bars represent ± SEM. Statistical analyses of the overall effects of the opioid agonists are presented in the legend to Figure.

## Abbreviations

WCS, Wooden Chest Syndrome; WHBP, Working Heart-Brainstem Preparation; EMG, Electromyography; BRET, Bioluminescence Resonance Energy Transfer; SGP, Subglottal Pressure; HEK 293, Human embryonic kidney 293 cells; DMEM, Dulbecco’s Modified Eagle’s Medium.
