# Supplementary material for: Comparison of the effects of fentanyls and other μ opioid receptor agonists on the electrical activity of respiratory muscles in the rat
Source: Front Pharmacol. 2023 Nov 23;14:1277248. doi: 10.3389/fphar.2023.1277248 (PMC10710149; doi:10.3389/fphar.2023.1277248)
Supplement: Supplementary file 1 [file DataSheet1.docx]

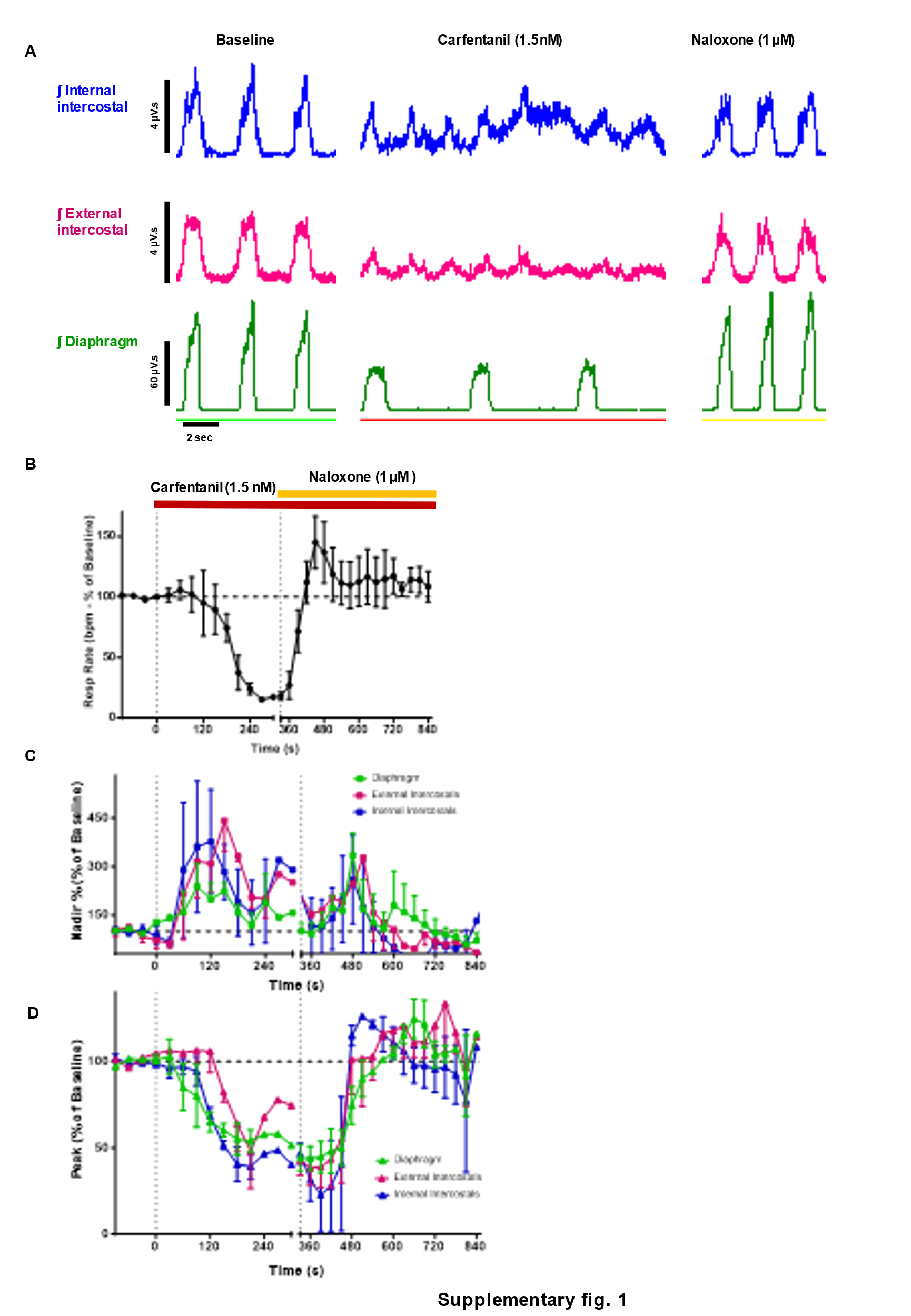


**Supplementary fig. 1.** **Effect of 1.5 nM carfentanil (n=3) on respiratory rate and on EMG activity in the diaphragm, external and internal intercostal muscles.** (**A**) Baseline integrated and raw EMG recordings before drug addition (left panel), during perfusion with carfentanil (1.5 nM) (middle panel) and following addition of naloxone (1 μM) to the carfentanil-containing perfusate (right panel). (**B**) Carfentanil (1.5 nM) depressed respiratory rate by 85%, that was fully reversed by naloxone (1 μM). (**C**) Carfentanil (1.5 nM) increased nadir EMG activity in the internal and external intercostal muscles, with a smaller effect in the diaphragm; naloxone (1 μM) didn’t fully reverse carfentanil-induced increase of nadir. (**D**) Carfentanil (1.5 nM) decreased peak EMG activity in all the respiratory muscles; naloxone (1 μM) reversed the carfentanil-induced decrease in peak but with a slower rate of onset than that observed for reversal of fentanyl (see Fig. 4). Data points were averaged in 30 second bins, and the subsequent change following drug administration calculated as a percentage of the pre-drug baseline. Error bars represent ± SEM. Statistical analyses of the overall effects of the opioid agonists are presented in Tables 1 and 2.
